# A survey of Big Data dimensions vs Social Networks analysis

**DOI:** 10.1007/s10844-020-00629-2

**Published:** 2020-11-09

**Authors:** Michele Ianni, Elio Masciari, Giancarlo Sperlí

**Affiliations:** 1 DIMES - Department of Informatics, Modeling, Electronics and Systems, University of Calabria, 87036 Arcavacata, CS Italy; 2 Department of Electrical and Information Technology (DIETI), University of Naples Federico II, via Claudio 21, 80125 Naples, Italy

**Keywords:** Big Data, Social Network, Centrality measure, Fake news

## Abstract

The pervasive diffusion of Social Networks (SN) produced an unprecedented amount of heterogeneous data. Thus, traditional approaches quickly became unpractical for real life applications due their intrinsic properties: large amount of user-generated data (text, video, image and audio), data heterogeneity and high speed generation rate. More in detail, the analysis of user generated data by popular social networks (i.e Facebook (https://www.facebook.com/), Twitter (https://www.twitter.com/), Instagram (https://www.instagram.com/), LinkedIn (https://www.linkedin.com/)) poses quite intriguing challenges for both research and industry communities in the task of analyzing user behavior, user interactions, link evolution, opinion spreading and several other important aspects. This survey will focus on the analyses performed in last two decades on these kind of data w.r.t. the dimensions defined for Big Data paradigm (the so called Big Data 6 V’s).

## Introduction

Since the initial definition of Big Data (Agrawal et al. 2012; Labrinidis and Jagadish 2012; IBM et al. 2011; Manyika et al. 2011; Lohr 2012), this paradigm has been driving both research and industrial communities. These kinds of data exhibit peculiar features as well as high volume, velocity, variety that pose quite intriguing problems for assessing their value and veracity (Noguchi 2011a, 2011b). Among the plethora of Big Data types, the ones pertaining social networks are very challenging (Easley and Kleinberg 2010) due to the large amount of produced heterogeneous data (i.e. multimedia, text and audio) and the velocity with which they are generated. The analysis of the interaction among users became quickly a crucial activity both for research community, that are devoted to address continuously new challenges. and for the companies, aiming to increase their revenues. A common problem for both scenarios concerns the validation of user generated content requiring proper elaboration to achieve actionable knowledge. As a matter of fact, the search for “Big Data” keyword on Scopus,^1^ a well reputed bibliographic indexing service, outputs 140*k* documents while the search for “Social Networks” retrieves 220*k* documents. There is a need for a more sophisticated retrieval approach for identifying those documents that could be interesting to analyze the Social Networks realm w.r.t. their Big data features. Indeed, while the analysis of Volume, Velocity, Variety and Variability could be just a bit simpler, dealing with Veracity and Value of information spreading over Social Networks is harder.

To better understand such a tie between Big Data research and Social Networks (SN) we briefly recall why SN are key Big Data providers. Indeed, SNs continuously generate an enormous quantity of heterogeneous data gathering the most valuable information: user behaviors. This unprecedented amount of data is leveraged by the “Do ut Des” strategy of the big companies (i.e. Amazon, Apple, Facebook, Google or Microsoft) who provide several services for free while gathering user data. In this respect, we briefly recall why SNs match perfectly the Big Data definition:^2^

- *Volume*: There are 4 Billion SN active users;
- *Velocity*: 5 Billion contents are posted every (2-5 new users per second) day;
- *Variety*: Posts contains texts, images, videos;
- *Variability*: Post contents is quite heterogeneous;
- *Veracity*: Contents has to be checked as they come mainly from not verified sources;
- *Value*: The market value is 20 Billion Dollar per year mainly spent for social media advertising.

Furthermore, other V’s have been defined for SNs:

- *Virality*: It refers to the wide use of re-posting that is an easy “cut and paste” strategy for sharing interesting information;
- *Viscosity*: It tries to evaluate how much the information diffusion triggers user reactions;
- *Visualization*: As the visual representation of information, intuitively, makes sense of a phenomenon and triggers (sometimes wrong) decisions.

For the sake of completeness, we mention here that such data are produced *directly* by users who decide to share almost everything (photos, comments, political opinions, recipes, food recommendations, travel positions, mood...) or *indirectly* by the social network providers who enrich the original user data by adding semantic meta data, statistics and usage patterns.

In order to avoid confusion, we must first distinguish between two common approaches: social media analytics (Newman 2010) and social network analysis. Both approaches had a great impact on the analysis of new communication models that came out after social network pervasive diffusion as they try to provide answers both to company and societal needs. More in detail, social media analytics (Shum and Ferguson 2012) refers to the process of analyzing the information exchanged by network users about products, brands or topics. In particular, the main difference between social media analytics and social network analysis concerns which data are used to support different applications (Zeng et al. 2010); in fact, the former relies on the analysis of heterogeneous data (i.e. tags, user-expressed subjective opinions, ratings, user profiles and both explicit and implicit social networks) whilst the latter is mainly based on the study of user-to-user relationships. In this field, we can mention the approaches for brand advocacy (Schepers and Nijssen 2018; Liu et al. 2017a), reputation management (Budak et al. 2011b; Liu et al. 2019), competitor analysis (Valera et al. 2014; Fu et al. 2019), community management (Dholakia and Vianello 2009), customer management (Mossel and Tamuz 2012), viral marketing (Lu et al. 2013b; Maurer and Wiegmann 2011; Trattner and Kappe 2013) and sentiment analysis (Mäntylä et al. 2018; Jiménez et al. 2019) to cite a few. The expected outputs of these analyses are information about: Why people like or dislike a product? Who are the top competitors online? What are the media that mainly deliver interesting information about a company? What are the features associated to a given brand? What are the sentiments about a political candidate? What are the trend topics? Answering the questions above can provide valuable information about company (target) users, that can drive important decisions like the timing of posting key information. More in detail, by analyzing user posts it is possible to find the best time in order to share new information, to better format them and wisely choose the best medium and strategy. To summarize, it is important to properly quantify the impact of the above mentioned information and to measure in terms of generated volumes, user reach and lead generation to design suitable tools aiming at revenue maximization when investing on social networks for business purposes. As a matter of fact, providing news and advertising when users are online and at their highest level of alertness can lead to effective engagement, higher traffic that, in turn, could increase company sales.

On the other side, social network analysis (Wasserman and Faust 1994) mainly focus on the ties among nodes in the network. In particular, the goal of this analysis is to gather information about the actual links of a given user (Beigi et al. 2016), how much the user is popular in the network (Brandtzaeg and Heim 2009), how much the user is central w.r.t. the network (Landherr et al. 2010), how the information flow across the network (Lou et al. 2014b; Bonchi et al. 2010; Tang et al. 2014b; Wang et al. 2012; Kempe et al. 2003b). It is straightforward to notice that, the wide variety of topics and their practical implications calls for a research effort that should involve multidisciplinary skills. Moreover, social network analysis can be classified by considering the level where the analysis take place, i.e., we could be interested in micro analysis that can be done with a subset of the social data of interest. By this kind of analysis, we can obtain recommendations based on user preferences, or if the target are small communities found in a large social network (Barbieri et al. 2017), how those communities trust each other (Costa et al. 2014), how people in each community interact (Cassavia et al. 2018) and become influencers for their peers (Bazzi et al. 2018) and eventual user non-progressive features. Finally, an increasing effort has been devoted to Fake news approaches (Bondielli and Marcelloni 2019a). On the other side, macro analysis mainly focus on large networks, where interesting measures like centrality (Kourtellis et al. 2016), or the use of algorithms, such as community search, are important.

### Survey organization

During the last decade the above mentioned problems have been addressed by several research groups that explored different research directions providing very interesting solutions. In this survey, we will try to give the reader an overview of the main approaches for social network analysis w.r.t. the big data features of these peculiar kind of data. More in detail, we will first give an introduction to social network analysis by discussing the basic concepts of social network topology (Ferrara and Fiumara 2011; Mossel et al. 2012) and social network indexes (Singh et al. 2017; Kang et al. 2011; Brandes et al. 2016). After this introduction, we will cover the big data aspects of social network analysis (Saleh et al. 2013) by describing their distinguishing features. Moreover, we will discuss the main approaches for behavioral analysis in social networks (Cassavia et al. 2017a; Amato et al. 2018; Laleh et al. 2018). Furthermore, we will show how to take advantage of social networks analysis in real life scenarios and some successful examples of systems leveraging the techniques previously described. Obviously, we will try to provide main references that can guide the reader through the plethora of papers published on the topics in the last decade. We point out that some dimensions like Veracity and Value have been studied extensively thus we will be able to provide more references, however we will try to cover each dimensions avoiding overlaps. In a sense, if an approach involve more than one dimension we will discus it w.r.t. the predominant one. We will not review the current commercial tools as this aspect falls beyond of the scope of this survey but we cite here some of the data analysis frameworks currently used for social network analysis (e.g., Centrifuge (http://centrifugesystems.com/), Commetrix (http://www.commetrix.de/), Cuttlefish (http://cuttlefish.sourceforge.net/), Egonet (https://sourceforge.net/projects/egonet/), Gephi (https://gephi.org/), InFlow (http://orgnet.com/index.html), etc.). These tools can be used as a starting point to figure out how to build a system tailored for managing Big Data features of SN.

The main novelties of this surveys can be summarized as follows:

- we analyze V’s dimension according to applications domain, focusing on pros and cons;
- we investigate how it is possible to extract high **V** alue from the large body;
- we examine data collection architectures for dealing with Volume and Velocity.

Finally, this survey has been designed for practitioners in social media analysis and big data, including experts in marketing, economics and social science interested in the analysis of social media information.

## Social Network basics

### Social Network topology

**Fig. 1:**
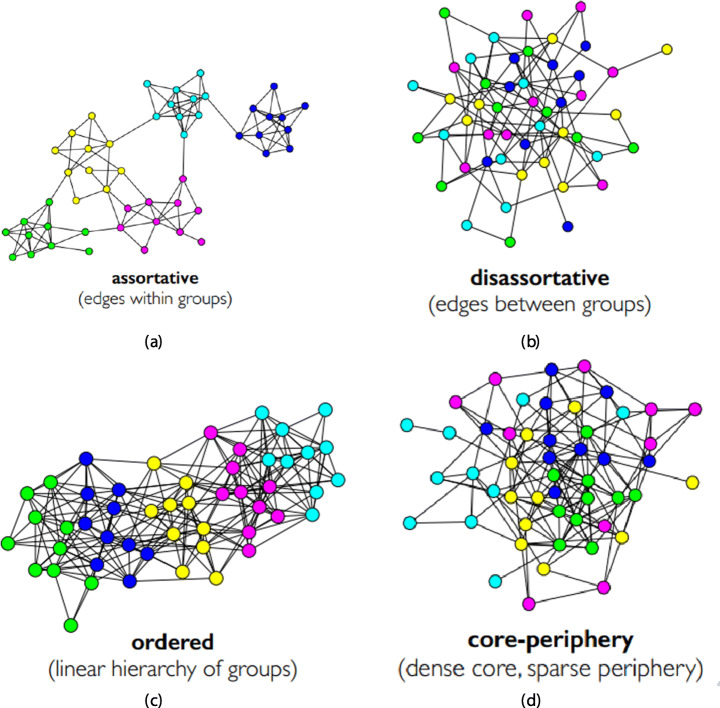
Network topologies

According to a widely accepted definition (Boyd and Ellison 2007) a social network is a group of individuals, e.g., friends, acquaintances, and coworkers that are connected by interpersonal relationships. This concept dates back till the beginning of human history (think about the ancient Greek squares) but in last decade the use of *online* services and the pervasive use of PC, tablet and mobile phones caused an exponential growth of social networks both in their scale and purposes. They differ from other network structures (biological, transport and telecom to cite a few) because of the presence of positive degree correlations named as *assortativity* (Fisher et al. 2017). Indeed, in social networks the similar behavior to group together has been defined as *homophily*, which implies, from a topological point of view, that there are many edges within a group of similar people on the network as shown in Fig. 1a. Examples of such behaviour can be found on Facebook, Twitter, Pinterest, Instagram, Reddit (Cauteruccio et al. 2020) to cite a few.

However, there exist also disassortative networks whose topology is characterized by edges between groups as reported in Fig. 1b (like for example in Tinder where in general people tend to establish a link with people of a different group). Some networks are hierarchical (see Fig. 1c), i.e. there is an ordering for groups (like Google+) while few of them are core-periphery shaped (see Fig. 1d) , i.e., there is a dense core connected to a sparse periphery (like Networks of Network scientists).

Indeed, social ties evaluation dates back earlier than internet and social networks came on scene. In Granovetter (1983) and Burt (1992), the authors outline the importance of Social Networks metrics^3^ like centrality measures (in particular betweenness centrality), thus, in what follows we briefly recall the main metrics used in literature along with some approaches that tackle the huge actual size of networks that poses many computational issues.

### Centrality measures

**Fig. 2:**
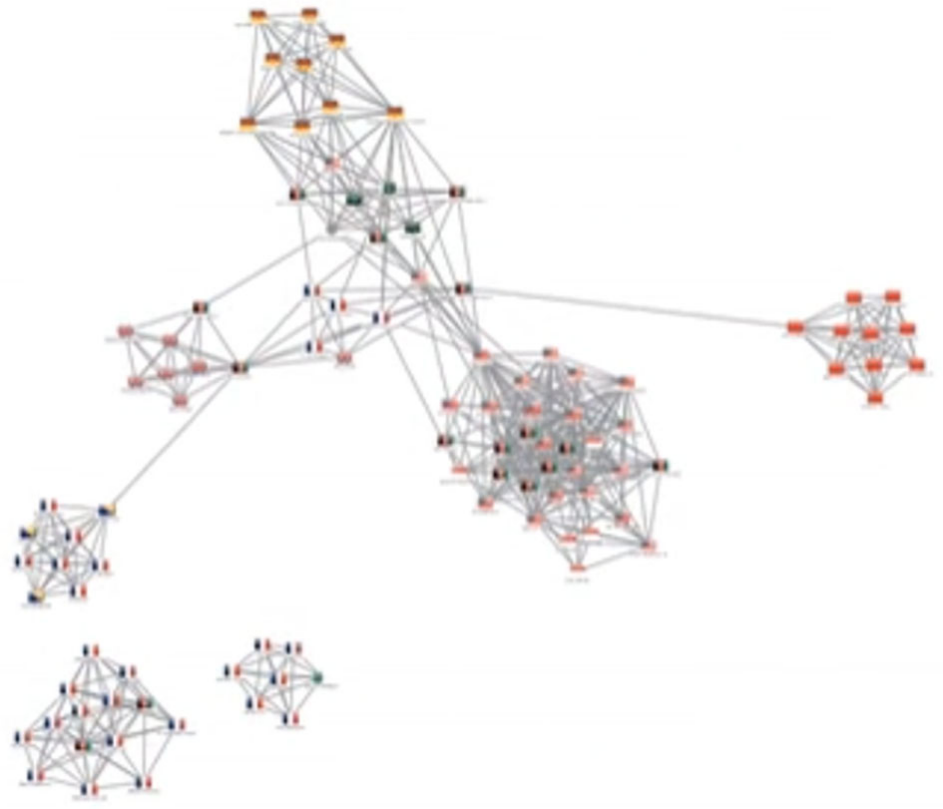
Degree centrality

**Fig. 3:**
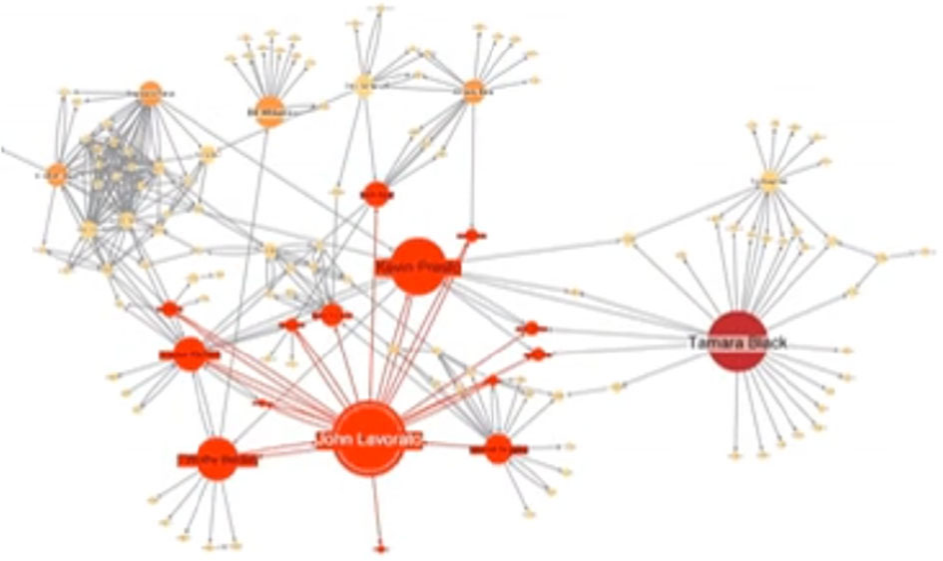
Betwenness centrality

**Fig. 4:**
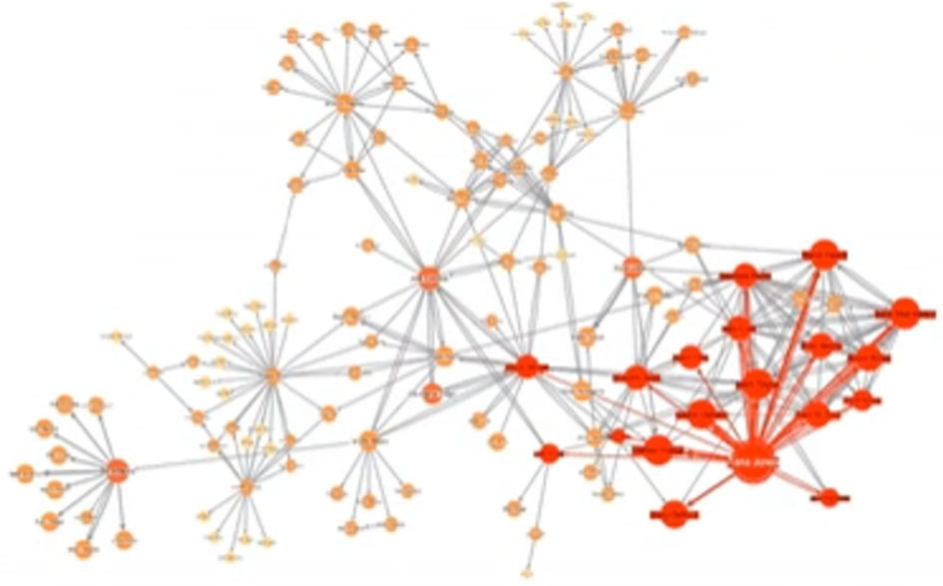
Closeness centrality

**Fig. 5:**
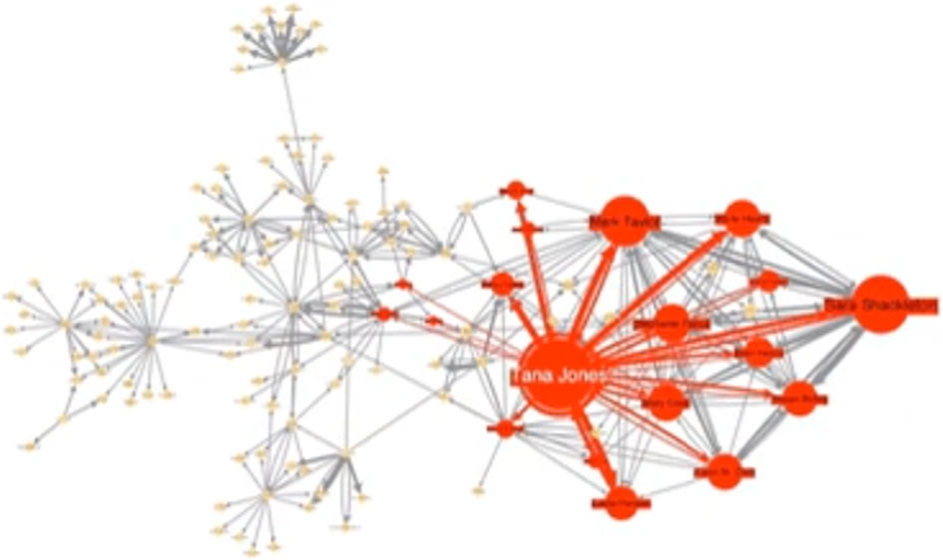
Eigen centrality

**Fig. 6:**
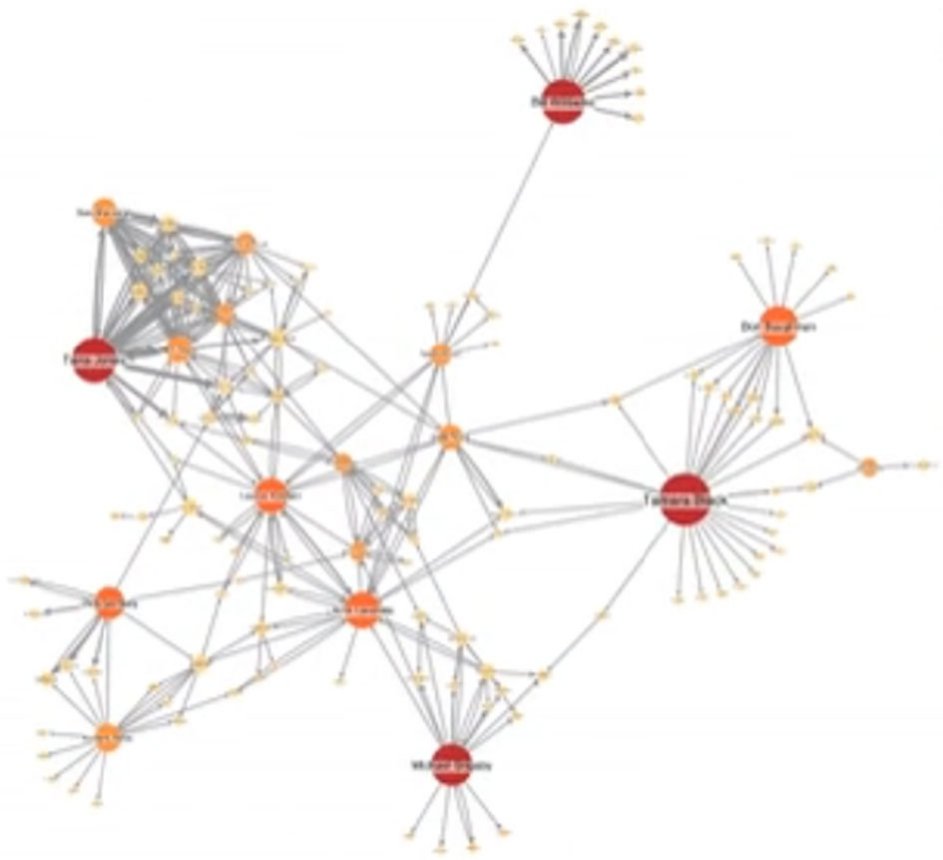
Page rank

In Figs. 2, 3, 4, 5 and 6 we summarize main social network centrality measures, that are widely used for networks analytics. For each measure we show a plot^4^ and the main information about their definition, usage and useful information that can be gathered to provide a quick guide to their understanding.

In Fig. 2, we show an example of degree centrality measures whose main characteristics are:

- **Definition**: Degree centrality assigns an importance score based purely on the number of links held by each node.
- **What it tells us**: How many direct, ‘one hop’ connections each node has to other nodes within the network.
- **When to use it**: to find strongly connected individuals, popular individuals, individuals who are likely to hold most information or individuals who can quickly connect to the wider network.
- **A bit more detail**: Degree centrality is the simplest measure of node connectivity. Sometimes it’s useful to look at in-degree (number of inbound links) and out-degree (number of outbound links) as distinct measures, for example when looking at transactional data or account activity.

An example of betweenness centrality has been shown in Fig. 3, where the largest nodes are those having higher centrality measurement values.

- **Definition**: Betweenness centrality measures the number of times a node lies on the shortest path between other nodes.
- **What it tells us**: This measure shows which nodes act as “bridges” between nodes in a network. It does this by identifying all the shortest paths and then counting how many times each node falls on one.
- **When to use it**: To find the individuals who influence the flow around a system.
- **A bit more detail**: Betweenness is useful for analyzing communication dynamics, but should be used with care. A high betweenness count could indicate someone holds authority over, or controls collaboration between, disparate clusters in a network; or indicate they are on the periphery of both clusters.

For large evolving scalable graphs online computation of betweenness centrality has to be performed by network vertices and edges taking into account edge addition and removal (Girvan and Newman 2002). In a recent paper a carefully engineered algorithm with out-of-core techniques tailored for modern parallel stream processing engines that run on clusters of shared-nothing commodity hardware has been presented and showed satisfactory performances (Kourtellis et al. 2016).

Furthermore we analyzed also the *Closeness* centrality, whose example has been depicted in Fig. 4. In the following, we investigate the main characteristics of *Closeness* centrality:

- **Definition**: This measure scores each node based on their “closeness” to all other nodes within the network.
- **What it tells us**: This measure calculates the shortest paths between all nodes, then assigns each node a score based on its sum of shortest paths.
- **When to use it**: To find the individuals who are best placed to influence the entire network most quickly.
- **A bit more detail**: Closeness centrality can help find good “broadcasters”, but in a highly connected network you will often find all nodes have a similar score. What may be more useful is using Closeness to find influencers within a single cluster.

An example of *EigenCentrality* has been depicted in Fig. 5, whose main characteristics are:

- **Definition**: Like degree centrality, EigenCentrality (Newman 2006; Wang et al. 2008) measures a node’s influence based on the number of links it has to other nodes within the network. EigenCentrality then goes a step further by also taking into account how well connected a node is, and how many links their connections have, and so on through the network.
- **What it tells us**: By calculating the extended connections of a node, EigenCentrality can identify nodes with influence over the whole network, not just those directly connected to it.
- **When to use it**: EigenCentrality is a good “all-round” SNA score, handy for understanding human social networks, but also for understanding networks like malware propagation.
- **A bit more detail**: it is possible to calculates each node’s EigenCentrality by converging on an eigenvector using the power iteration method.

In Fig. 6 it is shown an example of *EigenCentrality*, whose main peculiarities are:

- **Definition**: PageRank is a variant of EigenCentrality, also assigning nodes a score based on their connections, and their connections’ connections. The difference is that PageRank also takes link direction and weight into account - so links can only pass influence in one direction, and pass different amounts of influence.
- **What it tells us**: This measure uncovers nodes whose influence extends beyond their direct connections into the wider network.
- **When to use it**: Because it factors in directionality and connection weight, PageRank can be helpful for understanding citations and authority.
- **A bit more detail**: PageRank is famously one of the ranking algorithms behind the original Google search engine (the ‘Page’ part of its name curiously is the same of creator and Google founder, Larry Page).

Nevertheless, the computation of these measures is expensive in terms of running time as the number of nodes and, in particular, the number of arcs increases. For this reason, different approaches (You et al. 2017; Brandes 2004; García and Carriegos 2019) have been proposed to try to limit this problem. In You et al. (2017), the authors propose deterministic algorithms, which converge in finite time, for the distributed computation of the degree, closeness and betweenness centrality measures in directed graphs. They design distributed randomized algorithms to compute PageRank for both fixed and time-varying graphs. An interesting feature of the proposed algorithms is that they do not require to know the network size, which can be simultaneously estimated at every node, and that they are clock-free. In Brandes (2004), some algorithms for betweenness centrality computation that requires *O*(*n* + *m*) space and runs in *O*(*nm*) and *O*(*nm* + *n*2*logn*) time on unweighted and weighted networks, respectively, where *m* is the number of links have been presented. The authors proved that their approach substantially increases the range of networks for which centrality analysis is feasible. In García and Carriegos (2019), a parallel implementation in C language of some optimal algorithms for computing of some indicators of centrality has been proposed. More in detail, the parallel implementation heavily reduces the execution time of their sequential (non-parallel) counterpart. The proposed solution relies on threading, allowing for a theoretical improvement in performance close to the number of logical processors (cores) of the single computer in which it is running.

## Paper taxonomy

In order to provide the reader an easy way to orientate herself among the plethora of articles analyzed in this survey, we organized them in Table 1.

**Table 1:** Essential bibliography for every V

V dimension	Bibliography	Main application field
Veracity	García Lozano et al. (2020), Shao et al. (2020),	Fake news detection
	Bessi et al. (2015), Vicario et al. (2019),	
	Lazer et al. (2018), Li et al. (2019), Song et al. (2019),	
	Sharma et al. (2019), Kumar et al. (2016),	
	Shu et al. (2017), Rubin et al. (2015),	
	Bondielli and Marcelloni (2019a), Castelo et al. (2019),	
	Castillo et al. (2011), Ma et al. (2015),	
	Mihalcea and Strapparava (2009), Gilda (2017),	
	Khan et al. (2019), Jain and Kasbe (2018),	
	Kotteti et al. (2018), Hu et al. (2014), Zubiaga et al. (2016)	
	Wu et al. (2015), Ma et al. (2017), Hamidian and Diab (2019),	
	Kwon et al. (2013), Vosoughi et al. (2017),	
	Wang and Terano (2015), Gravanis et al. (2019),	
	Reis et al. (2019), Silva et al. (2020)	
Variety	Liu et al. (2012, 2017a), Erickson (2003),	Influence analysis
	Shen et al. (2006), Agreste et al. (2014),	
	Corradini et al. (2020), Wang et al. (2019),	
	Fang et al. (2014), Hamzehei et al. (2016), Lu et al. (2019),	
	Chen et al. (2015), Min et al. (2020),	
	Tian et al. (2020), Kalanat and Khanjari (2019),	
	Ianni et al. (2018, 2020)	
Variability	Barbieri et al. (2011b), Jackson and Rogers (2007),	Behavioral analysis
	Jacobs et al. (2017), Krivitsky and Butts (2017),	
	Clifton et al. (2004), Fernandez-Basso et al. (2019),	
	Ahmad et al. (2017), Persico et al. (2018),	
	Shi et al. (2018), Wu et al. (2018),	
	Zhang et al. (2017), Subbian et al. (2016),	
	Cassavia et al. (2017b)	
Volume and velocity	Anagnostopoulos et al. (2008),	System design
	Cassavia et al. (2017b), Mgudlwa and Iyamu (2018)	
Value	Liu et al. (2017a), Lin et al. (2015), Yuan et al. (2018),	Artificial Intelligence
	Bonchi et al. (2011), Anagnostopoulos et al. (2008),	
	Aral et al. (2009), Crandall et al. (2008),	
	Myers et al. (2012), Domingos and Richardson (2001),	
	Richardson and Domingos (2002), Kempe et al. (2003b),	
	Barbieri et al. (2011a, 2011b, 2012, 2014), Bhagat et al. (2012),	
	Aslay et al. (2016), Budak et al. (2011b),	
	Lu et al. (2013b), Tang et al. (2014b),	
	Lou et al. (2014a), Fang et al. (2019), Jannach et al. (2016)	
	Li et al. (2007), Cao et al. (2019),	
	Cassavia et al. (2018)	

This table summarizes all the relevant papers we cited (that are described in more details in the following sections) for each dimension of analysis and we report in the third column the main application filed. We choose to specify the scenarios exhibiting the higher number of papers related to the dimension being considered. This choice is not a limitation but on the contrary it aims to provide a quick pick suggestion when trying to study a specific dimension or phenomena.

## Dealing with volume and velocity

In this section we will discuss some approaches that have been leveraged in order to address the problem of collecting huge amount of social network data that exhibit an high velocity in their arrival rates. We will discuss them together as it is not convenient to decouple the data acquisition from the data storage steps. We first give a quick evaluation of the general problem of dealing with huge volume and fast data production by showing in Table 2 a snapshot of main SN features w.r.t. **V** olume and **V** elocity.^5^ As it is easy to see a system that would like to take advantage of these information should be carefully designed.

**Table 2:** A snapshot of some SN statistics

S.No	Source of data	No. of elements	Frequency
1	Tweets	More than 9,000	Per Second
2	Facebook Updates	More than 41,000	Per second
3	Emails	2,398,54 Mails	Per second
4	Google Search	More than 40,000	Per second
5	Youtube	101,604 Videos	Per second
6	Instagram	2,000+ Photos	Per second
7	Tumblr	More than 1964 Posts	Per second
8	Skype	More than 1,700 Posts	Per second

**Fig. 7:**
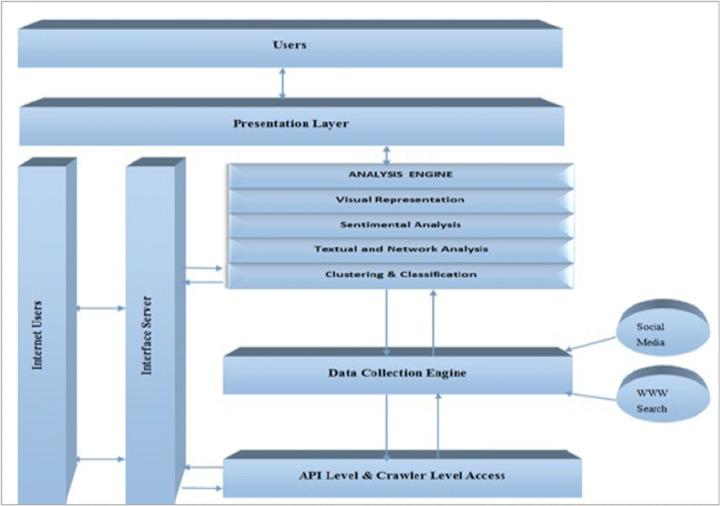
A system for data collection

Obviously, there is a limit to the accessible data for most of the companies and projects, thus we treat this aspect of big data and SN as a methodological problem rather than a “pure” research one. Indeed, many systems try to leverage tools like Interface Server, API level Access, Data Collection Engine and Analysis Engine (Kumar and Rishi 2015) like shown in Fig. 7.

**Fig. 8:**
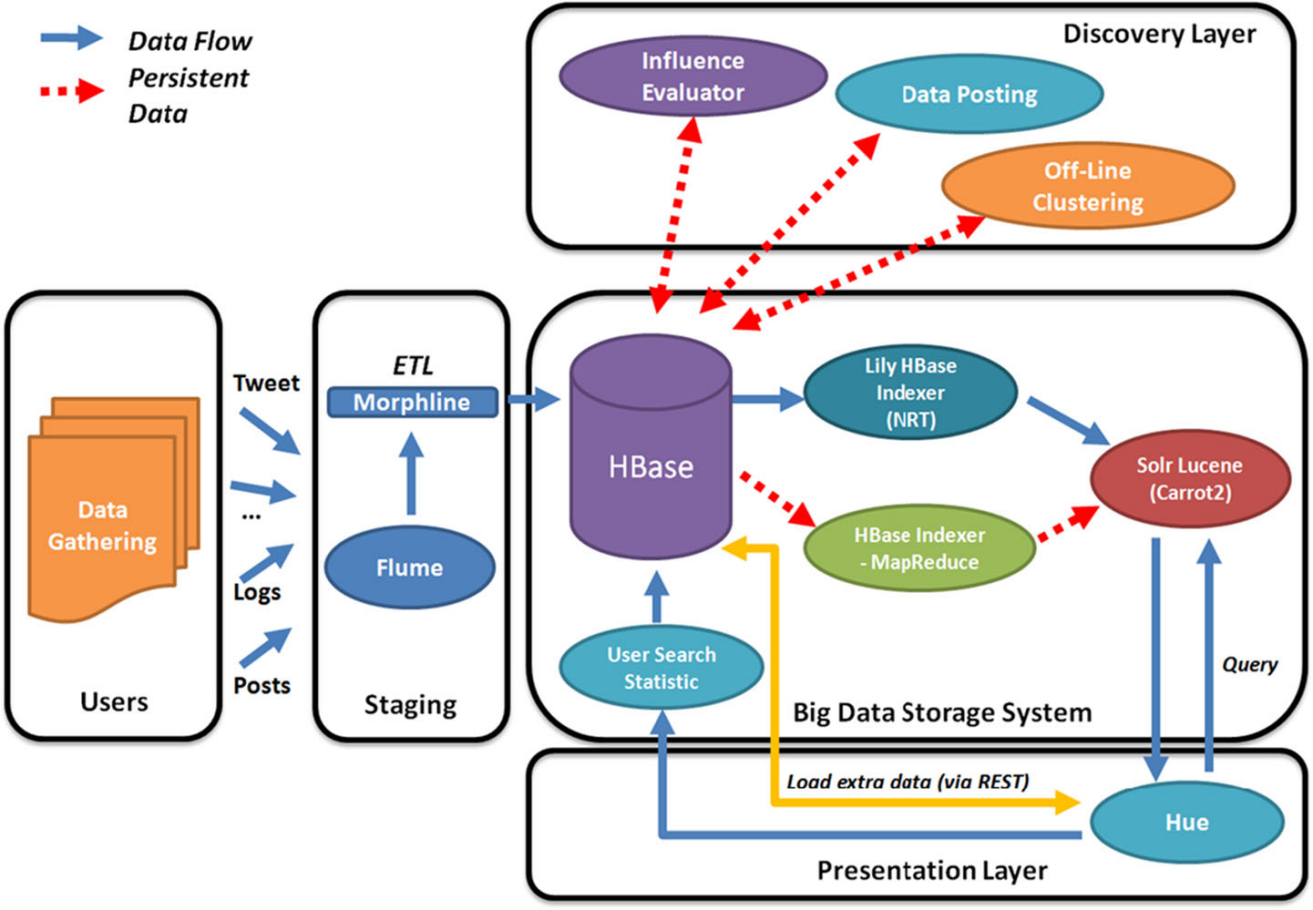
A system for data collection and transformation

In some cases, it is more convenient to store a fraction of the incoming data after a pre-elaboration step that can be performed by leveraging tools like the ones offered by Cloudera suite (Cassavia et al. 2017b) like shown in Fig. 8. The latter choice is preferable when the goal is a quick and less accurate online analysis of data while postponing a deeper one.

**Fig. 9:**
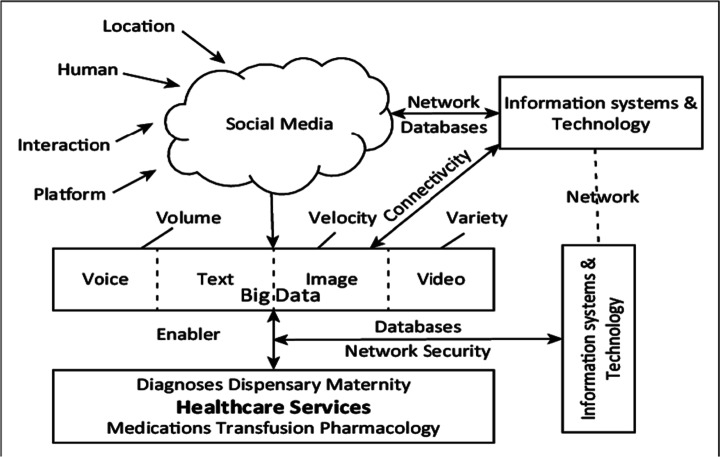
A system for SN and Big Data collection for Healthcare

The collection of Big Data pertaining to Social networks has also been used for improving e-health services. In Mgudlwa and Iyamu (2018) (the presented system is shown in Fig. 9), the authors examine the possible outcome of a better understanding of the complexities that are associated with the use of social media and healthcare big data, through influencing factors and implement the framework reported in Figure

## How to extract value

The most intriguing aspect of leveraging Big Data approaches for SN is the possibility to get high **V** alue form the large body of user generated contents (UGC) as they are a continuous source of profitable information. As a matter of fact UGC comes from a variety of venues, such as tweets or Facebook pages, pictures (e.g., Pinterest), blogs, microblogs, and product reviews (e.g., Amazon, Yelp). Empirical findings show that UGC has significant effects on brand images, purchase intentions and sales (an important role is played by ‘@’ and hashtags). However, due to their unstructured nature, it is important to leverage advanced modeling to properly identify sentiments that have a marketing value. In this respect one of the most effective social is Twitter as shown in Liu et al. (2017b) where the authors propose a framework that automatically derives brand topics and classifies brand sentiments. They explore the brand-related questions on Twitter by applying both LDA and sentiment analysis to 1.7 million tweets, moreover they leverage benchmarking against ACSI and expertise from industry experts. A similar analysis have been performed for Weibo in Lin et al. (2015) where a microblog-oriented sentiment lexicon is built and a lexicon-based sentiment orientation analysis algorithm is designed to classify sentiments.

Recently, social network formation have attracted increasing attention from both physical and social scientists. In Yuan et al. (2018) the authors presented a study on network embedding algorithms in machine learning literature that consider broad heterogeneity among agents while the social sciences emphasize the interpretability of link formation mechanisms. Thus, they define a social network formation model that integrates methods in multiple disciplines and retain both heterogeneity and interpretability by leveraging “endowment vectors” that encapsulates agents features and game-theoretical methods to model the utility of link formation.

On the business side, APQC’s Process Classification Framework (PCF) serves as a high-level, industry-neutral enterprise process model that allows organizations to see their business processes from a cross-industry viewpoint. In Bonchi et al. (2011) the authors propose a nice classification of business process w.r.t the SN area of expertise needed to improve them.

One of the most challenging problems for getting value from SN is the identification of which users are susceptible, how do information diffuse and if a company/user can trust other user opinion. Tese information are crucial as users continuously perform actions like posting messages, pictures, video, buying goods, expressing comments while they are connected with other users, thus interacting and possibly causing influence to spread. In this respect we must distinguish between *homophily*, i.e., the tendency to stay together with people similar to you (“Birds of a feather flock together”) and *social influence*, i.e., a force that person A (namely the influencer) exerts on person B to introduce a change of the behavior and/or opinion of B (influence is a causal process). In this area the main problem is to distinguish social influence from homophily and other factors of correlation as deeply discussed in Anagnostopoulos et al. (2008) and Aral et al. (2009).

In Crandall et al. (2008), the authors develop techniques for identifying and modeling the interactions between social influence and selection, using data from online communities where both social interaction and changes in behavior over time can be measured. In Myers et al. (2012) the authors used Twitter traces to study how information reaches the nodes of the network. They quantify the external influences over time and describe how these influences affect the information adoption. As an outcome they found that the information tends to “jump” across the network, which can only be explained as an effect of an unobservable external influence on the network. The social influence evaluation is quite important for the possible outcome of accurate predictions. Indeed, social influence can be very effective for marketing. In this field, the basic assumption is to leverage the word-of-mouth effect, thanks to which actions, opinions, buying behaviors, innovations and so on, propagate in a social network. More in detail, the goal is to target users who are likely to produce the above mentioned word-of-mouth diffusion, thus leading to additional reach, clicks, conversions, or brand awareness. The first problem of this kind to be studied was how to find a seed-set of influential people such that by targeting them it is possible to maximize the spread of viral propagations (Domingos and Richardson 2001; Richardson and Domingos 2002; Kempe et al. 2003a). After those initial attempts researchers focused on topic-aware Social Influence Propagation Models in order to better target users and differentiate them. As an example, in Barbieri et al. (2012), the authors introduce a topic-aware influence-driven propagation models that proved to be more accurate in describing real-world cascades than the standard (i.e., topic-blind) propagation models studied in the literature. In particular, they propose topic-aware extensions of the well-known Independent Cascade and Linear Threshold models.

Furthermore, classical diffusion models such as Independent Cascade and Linear Threshold do not distinguish between influence and product adoption as they implicitly assume that once influenced, a node necessarily adopts a product and that adopters always influence other users to adopt the product. Sometimes influenced users, once they become active, may choose to not adopt but instead tattle about the product; by doing so, they may either promote or inhibit adoption by other users A propagation model called LT-C model that accounts for these observations for modeling product adoption has been presented in Bhagat et al. (2012).

Another important problem is the evaluation of rewarding strategies for influential users. In Aslay et al. (2016) a model that allows influential user of a SN to get some money on the advertising revenue is presented. In particular, the study on incentivized social advertising formulate the problem of revenue maximization from the host perspective, when the incentives paid to the seed users are determined by their demonstrated past influence in the topic of the specific ad. The authors present two greedy algorithms for the problem: CA-GREEDY is agnostic to users’ incentives during the seed selection while CS-GREEDY is not.

In some scenarios, there exists competing campaigns in a social network (Multi-Campaign Independent Cascade (MCICM)). In that case, the problem is influence limitation when a “bad” campaign starts propagating from a certain node in the network. In Budak et al. (2011a) the authors introduce the notion of limiting campaigns to counteract the effect of misinformation. The latter is performed by identifying a subset of individuals that need to be convinced to adopt the competing (or “good”) campaign so as to minimize the number of people that adopt the “bad” campaign at the end of both propagation processes.

An interesting problem arise when two or more players compete with similar products on the same network (competitive viral marketing). Here, from the host’s perspective, it is important not only to choose the seeds to maximize the collective expected spread, but also to assign seeds to companies so that it guarantees the “bang for the buck” for all companies is nearly identical. A solution is presented in Lu et al. (2013a) by introducing Needy Greedy, a propagation model that captures the competitive nature of viral marketing.

Sometimes, it is important to consider the magnitude of influence and the diversity of the influenced crowd simultaneously. In this case, it is crucial to construct a class of diversity measures to quantify the diversity of the influenced crowd. In Tang et al. (2014a) the authors formulate this problem as an optimization one called diversified social influence maximization. Finally, we mention the case of non-progressive phenomena. Indeed, a user of a social network may stop using an app and become inactive, but again activate when instigated by a friend, or when the app adds a new feature or releases a new version. Here, the problem is that the progressive model for influence maximization is no more valid. In Lou et al. (2014a) influence propagation is modeled as a continuous-time Markov process with 2 states: active and inactive and compute the current state accordingly.

Another challenging problem to be considered is the discovery of communities within the networks. As a matter of fact, individuals tend to adopt the behavior of their social peers, so that cascades happen first locally, within close-knit communities, and become global “viral” phenomena only when they are able cross the boundaries of these densely connected clusters of people (Fang et al. 2019). An interesting approach is presented in Barbieri et al. (2014) where the distinction between common identity and common bond theory is elaborated. More in details, identity-based attachment holds when people join a community based on their interest in a well-defined common topic while bond-based attachment is driven by personal social relations with other specific individuals. Datta and Adar (2019) investigated Reddit social platform for unveiling inter-community conflicts. Furthermore, political communities (Soliman et al. 2019) have been investigated for supporting moderators.

Finally, we mention here the recommendation problem. More in details, the assumption that nowadays having more choices leads to more freedom and (thus) greater welfare is wrong. One of the side effects of so much choice is that it increases the likelihood that a user make a “wrong” choice, and the corresponding likelihood that s/he will regret her/his choice. Thus, Recommender Systems (RS) are reshaping the world of e-commerce, helping customers find and purchase products, such as songs, books, movies, or news with the aim of transforming a regular user into a buyer. Moreover, as the volumes of information continuously grow, the importance of RS is likely to continue to grow and to play a key role in many different industry domains. The prediction problem can be formulated as a matrix completion one (Jannach et al. 2016) in order to predict the value of the missing entries among the user preference data for building a recommendation list or can be modeled by probabilistic mixture models by choosing the most suitable one for the context being analyzed (Li et al. 2007; Cao et al. 2019; Barbieri et al. 2011a, 2011b).

## Dealing with veracity

We started the analysis of the Big Data dimensions from the **V** eracity as the extensive use of social networks for information sharing is causing an exponentially increase of user-generated content (i.e. videos, images or review). In particular, several challenges arose about fake news, malicious rumors, fabricated reviews, generated images and videos, that, nowadays, are manually or semi-automated verified (see García Lozano et al. 2020 for more details). For this reason, it is important to design automatic systems capable of verifying user-generated content, that typically are based on Artificial Intelligence techniques. In particular, truth discovery process is one the main issue in social network analysis because it requires to combine features extracted from different types of content with propagation analysis (as shown in Shao et al.^2020^).

In the last years, we are facing with a dramatic increase of Fake News (while writing this paper the world is facing a tremendous pandemia due to COVID-19 and the misinformation is worsening some social problems). Interestingly enough it has been shown that people tend to believe to false news as they tend to meet the user expectations and beliefs. In fact, researchers are recently focusing their attention on the analysis of misinformation, that are information perceived as inaccurate or misleading (Lazer et al. 2018), in the social network as well as Facebook (Bessi et al. 2015; Vicario et al. 2019), Twitter (Li et al. 2019) and so on. Different types of misinformation can be disseminated over social networks that can be classified in rumors (Song et al. 2019), fake news (Sharma et al. 2019) and hoaxes (Kumar et al. 2016). Based on this premise, several approaches have been proposed so far, in what follows we mention some of the most recent ones. Indeed, the fake news notion has evolved over the time assuming nowadays the sense of any article or message propagated through media platforms carrying behind it false or misleading information (Sharma et al. 2019). Some well known examples of fake news across history are mentioned below:

- During the second and third centuries AD, false rumours were spread about Christians claiming that they engaged in ritual cannibalism and incest;^6^
- In 1835 The New York Sun published articles about a real-life astronomer and a fake colleague who, according to the hoax, had observed bizarre life on the moon;^7^
- More recently we can cite some news like, Paul Horner, was behind the widespread hoax that he was the graffiti artist Banksy and had been arrested; a man has been honored for stopping a robbery in a diner by quoting Pulp Fiction; and finally the great impact of fake news on the 2016 U.S. presidential election, according to CBS News.^8^

Thus, fake news deceive people by creating a false impression or conclusion (Lazer et al. 2018) whose detection is made difficult by the use of heterogeneous topics and different linguistic styles for their production (Shu et al. 2017). Rubin et al. (2015) organized the fake news into three categories: *serious fabrications*, being prototypical form of fake news that often become viral through social media, *large scale hoaxes*, representing false information disguised as proper news, and *humorous fakes*, having the aim to amuse readers.

According to Bondielli and Marcelloni (2019a), it is possible to classify approaches for fake news detection on the basis of the exploited features into *content* and *user*-based techniques. The former has the aim to classify news according to their inherent content (mainly news text) (Castelo et al. 2019), whilst the latter aims to deal with dynamic propagation of fake news according to user-based, text-based, propagation-based and temporal-based features (Castillo et al. 2011; Ma et al. 2015).

The content-based approaches try to classify news according to their inherent content (mainly news text). Several machine learning methods have been then proposed for analyzing information content and performing the related classification. Nevertheless, it is frequent to observe a performance slump because classical classifiers are not able to generalize and to classify instances never seen before as, instead, it can happen for fake news.

The most effective content-based methods rely on the *N*-grams, i.e. sequences of *N* contiguous words within a text (e.g., unigrams, bigrams, trigrams etc.). The first interesting approach leveraging such kind of features has been proposed by Mihalcea and Strapparava (2009) for lie detection using Naïve Bayes and SVM classifiers in order to identify people’s lies about their belief. More recently, Gilda (2017) analyzed 11,000 articles from several sources applying term frequency-inverse document frequency (TF-IDF) of bi-grams within a probabilistic context free grammar (PCFG) for fake news detection. The evaluation has been performed using different classification methods as Support Vector Machines, Stochastic Gradient Descent, Gradient Boosting, Bounded Decision Trees, and Random Forests. A very useful work is that proposed by Khan et al. (2019), where they studied the performances of different content-based approaches on various datasets, evaluating also several features as well as lexical, sentiment and *N*-grams ones. In turn, Jain and Kasbe (2018) proposed a specified method based on Naive Bayes classifiers with the aim to predict if a given post on Facebook is real or fake. Finally, in Kotteti et al. (2018) the authors tried to handle the missing values problem in fake news datasets by using data imputation for both categorical, with the most frequent values in the columns, and numerical features, using the mean value of the related column. In addition, TF-IDF vectorization was applied in feature extraction to unveil main features to use as input for a Multi-Layer Perceptron (MLP) classifier.

In turn, user-based features are typically used for classifying users in genuine or fake (Hu et al. 2014) that could be used as measure of the reliability of the shared information. Other features concerns information about social circles and activities made in Online Social Media, as well as number of posts, following/follower or their ratio (Zubiaga et al. 2016), or account’s age and/or linking to external resources (Wu et al. 2015; Zubiaga et al. 2016). Nevertheless, information about user’s activities on Online Social Networks cannot typically be gathered due to privacy constraints. According to Ma et al. (2017) different studies rely on network-oriented features for analyzing diffusion patterns (Ma et al. 2015; Hamidian and Diab 2019) and modeling the temporal characteristics of propagation (Kwon et al. 2013).

Finally, some approaches (Vosoughi et al. 2017; Wang and Terano 2015; Wu et al. 2015; Ma et al. 2015) have been proposed combining content and user based features for fake news detection. As an example, Castillo et al. (2011) proposed a machine learning approach based on decision tree model for classifying news as fake combining three different types of features: user-based (e.g. registration age and number of followers), text-based (e.g. the proportion of tweets that have a mention ‘@’ ), and propagation based (e.g. the depth of the re-tweet tree).

Concerning benchmarks, different studies (Gravanis et al. 2019; Reis et al. 2019; Silva et al. 2020) have been designed to compare proposed approaches for fake news detection but they focused on small datasets and/or analyzed only some machine learning approaches.

Nevertheless, the majority of these approaches are based on supervised learning whilst fake news are generally related to newly emerging, time-critical events, which may not have been properly verified by existing knowledge bases due to the lack of confirmed evidence or claims. Furthermore, the content of fake news exhibits heterogeneous topics, styles and media platforms, aiming to mystify truth by diverse linguistic styles.

## The role of Variety in SN

**V** ariety is crucial in social media marketing as the potential target population in most application scenarios exhibits a lot of different behaviors within it. As a matter of fact, diversity characterizes how diverse a given user in a SN connects with its peers. In Liu et al. (2012), the authors give a comprehensive study of this concept. they propose some criteria to capture the semantic meaning of diversity, and then propose a compliant definition which is simple enough to embed this crucial concept.

In this section, we will start by briefly discussing the role of variety in human relationships as this will affect the analysis of this aspect at big data scale. More in detail, people are healthier and happier when they have intimates who care about and for them, but they also do better when they know many different people casually: acquaintance is important for better job (both finding and performing), indeed it is better than close ties (as mentioned in previous section this phenomenon is called the “The strength of weak ties”). As a matter of fact, wealthier people have diverse networks and acquaintance diversity also contributes to being better informed about health, politics and many other topics. People with wider networks are better informed about most things, but they may not realize how many of their good health practices go back to a thousand tiny nudges from casual conversations (Erickson 2003). Based on this premise we review in this section a bunch of approaches that try to evaluate the role of variety that is the less obvious and evident of the Big data features.

Since the initial diffusion of SN it has been clear that in order to have a quick understanding of the data variety it could be quite useful to leverage visualization approaches. In a sense variety can be considered as the set of “choice” individual can have, thus to tackle heterogeneous features ontology can be leveraged. In this respect, visualization is powerful as shown in Shen et al. (2006). The authors, used structural abstraction for implementing the Ontovis system that uses importance filtering to make large networks manageable and to facilitate analytic reasoning.

Another scenario where variety plays an important role is the human and cultural objects interaction. More in detail, correlation could emerge across the different activities a user can take part while interacting with art manufacts.

A nice study on aNobii, a social platform with a world-wide user base of book readers, who like to post their readings, give ratings, review books and discuss them with friends and fellow readers has been analyzed in Agreste et al. (2014) In particular, they analyzed the variety of roles by considering three subset of interaction: i) part social network, with user-to-user interactions, ii) part interest network, with the management of book collections, and iii) part folksonomy, with books that are tagged by the users. The obtained outcomes consists in an accurate user profiling that cannot be reduced to considering just any one type of user activity (although important): it is crucial to incorporate multiple dimensions to effectively describe users preferences and behavior. In order to reach this conclusion the authors carried out an experimental analysis by means of Information Theory tools like entropy and mutual information suggesting that tag-based and group-based profiles are in general more informative than wishlist-based ones. Finally, we will discuss the effect of variety on social influence. When social networks are heterogeneous (consisting of heterogeneous objects such as users, groups, and blogs), the influence they excerpt each other is affected by different types of objects on different topics (e.g., entertainment, marketing, and research).

An analysis of social commerce has been discussed in Nakayama and Wan (2019), where the authors analyzed how reviews made on these platforms (i.e. Yelp or Foursquare) affect users’ chooses. Furthermore, Chang and Li (2019) investigated social commerce (Yelp) to predict business performance of business object. In Corradini et al. (2020) the authors introduce the concept of *k-bridge*, a user who connects *k* sub-networks of a network or *k* networks of a multi-network scenario. This concept have important application in the analysis of opinion transmission, user influence and dissemination of information. The importance of *k-bridges* has been proved by analyzing the social network Yelp.

In Wang et al. (2019) the authors propose a context-aware user preferences prediction algorithm for location recommendation on LBSNs, using a dataset from Foursquare.

Topic-level influence mining has been investigated in Liu et al. (2012) by a generative model which utilizes both content and link information to mine direct influence strength in heterogeneous networks. Moreover, diffusion models for conservative and non-conservative influence propagations to learn indirect influence in social networks has been leveraged and the study has been validated against four different types of data sets extracted by different SN like Twitter, Digg, Renren and Cora.

The analysis of topics in the influence diffusion process allows to improve the advertisement injection phase according to different requirements. Fang et al. (2014) desigend a Topic-sensitive influencer mining (TSIM) by using an hypergraph learning in interest-based social media networks. A topic-based Social Measurement has been proposed by Hamzehei et al. (2016) on the basis of network structure, user-generated content and users interactions. Lu et al. (2019) measured topical users influence in OSN by combining users’ social relationships, posting and forwarding behaviors and posts content. A topic-aware influence maximization problem has been investigated by Chen et al. (2015) focusing on efficiency of algorithms keeping a high influence spread. Furthermore, time sensitivity has been also considered in Min et al. (2020) jointly considering topic preference and interaction delay. In turn, a deep reinforcement learning-based approach has been proposed by Tian et al. (2020) with the aim to identify a seed set of users that maximizes influence under query topics and diffusion model. In Kalanat and Khanjari (2019) the authors provide a technique to extract cost-effective actions which are formulated as an optimization problem where the objective is to learn actions consisting of the changes in the network that are likely to result in desired changes in the labels of intended individuals while minimizing the cost of the changes.

Finally, social data streams have been also investigated for improving the analysis of topic influence diffusion. In Chen et al. (2017), the authors analyzed social stream data to track dynamic users’ preferences for interpersonal influence. Furthermore, textual streams content has been investigated by Li et al. (2016) to deal with dynamic social networks. Zheng et al. (2020) developed a Topic-Specific Influence in Social Networks analyzing streaming data by using LSTM and self attention.

## Analyzing SN Variability

**Fig. 10:**
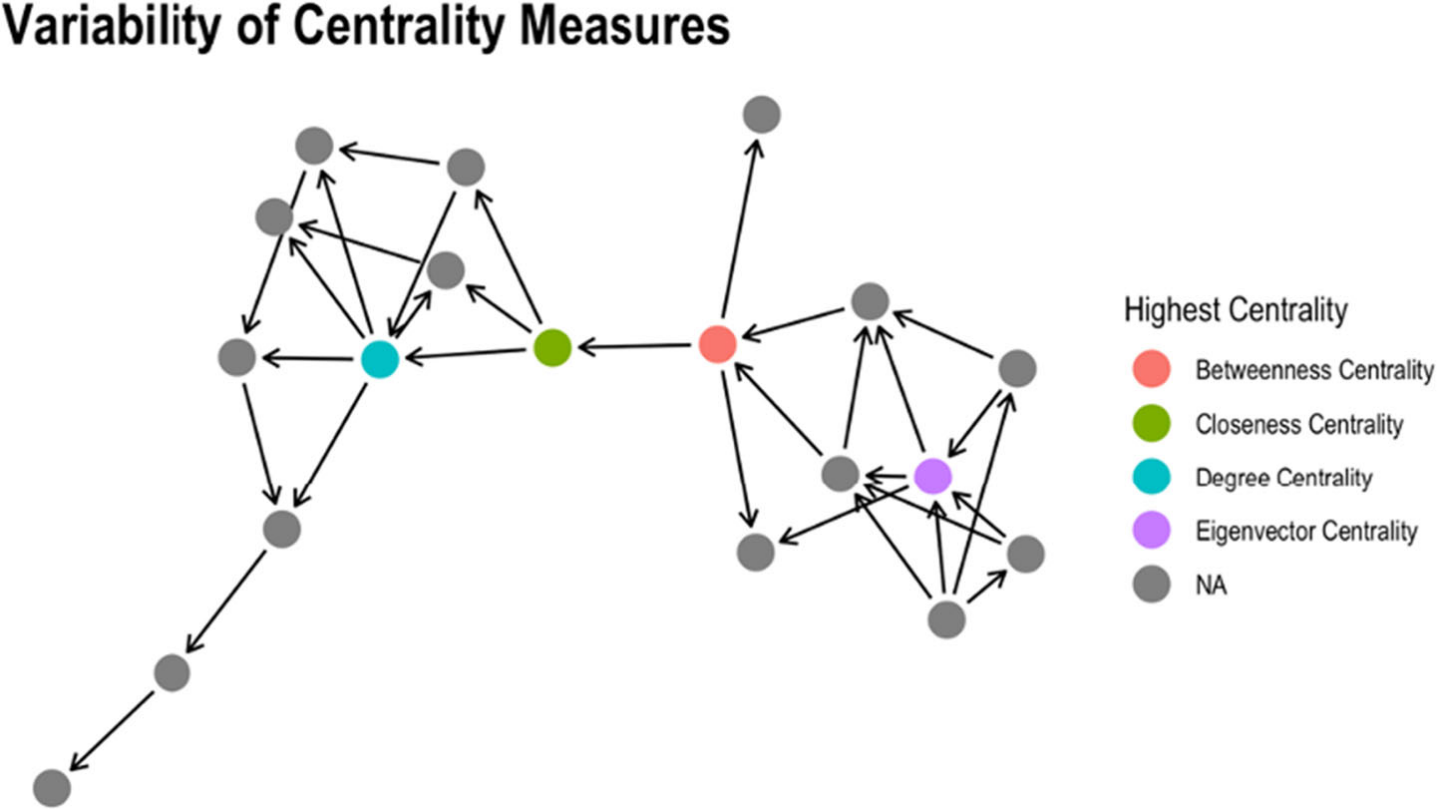
A snapshot of some SN statistics

Social groups present high **V** ariability due to age distribution individual features, gender, Interests (as they may change over time), personality, geographical distribution. Thus, the problem has to be tackled with a mix of sociology, economy, psychology skills aside the mere computer science ones. We also point out that information scale for this dimension is a severe limit to complete analysis as centrality measures are themselves highly variable as depicted in Fig. 10.

In Atalay (2013) the authors investigate the motivation for participants of social networks skewness, i.e., some of them have many contacts, while most others have few. They analyze the importance of age and randomness in explaining the variation in the number of contacts (i.e., the degree) that participants have and the underlying process that produces the degree distributions that are repeatedly observed in studies of social networks. Their work is based on Jackson and Rogers framework for network formation (Jackson and Rogers 2007) for building a model that allows nodes to differ in the rate at which they can expect to gain additional links. The fitness of a node is defined as the probability that each of its meetings will generate a link based not only on in-cohort features. They conclude that with more variability in fitness, there is more variability in the degree distribution of nodes belonging to a particular age.

Recently, scientists have deeply investigated the social networks influence on adolescents’ substance use behavior. Interestingly enough the influence varies by gender but the role of gender in this mechanism of influence needs further investigations. More in detail, the role an adolescent’s gender, alongside the gender composition of his/her network, plays in teenager approach to alcohol use is far to be fully understood. A study reported in Jacobs et al. (2017) investigated the associations among the gender composition of adolescents’ networks, select network characteristics, intra-personal, inter-personal factors and alcohol use for a sample of US adolescents. The authors performed cross-sectional data from a 2010 study of 1,523 high school students from a school district in Los Angeles. The analyses of adolescents’ network characteristics were conducted using UCINET 6 while logistic regression analyses testing the associations between gender composition of the network and alcohol use were conducted using SPSS 20. The reported results indicate that the gender composition of adolescents’ networks is associated with alcohol use. Adolescents in predominantly female or predominantly male friendship networks were less likely to report alcohol use compared to adolescents in an equal/balanced network. Additionally, depending upon the context/type of network, intrapersonal and interpersonal factors varied in their association with alcohol use. An important source of variability in SN data is geography. More in detail, geographical variability have potential implications on the structure of social networks. A study presented in Butts et al. (2012) demonstrate that geographical variability produces large and distinctive features in the “fabric” that overlies it. Many aggregate network properties can be fairly well predicted from relatively simple spatial demographic variables while spatial variability exert substantial influence on network structure at the settlement level. Moreover, spatial heterogeneity induce substantial within network heterogeneity, however geography drives many aggregate network properties in a predictable way.

Finally, we discuss the role of personality variability that represent another important factor to be evaluated. In particular, in Clifton (2013) the authors investigated how the contextual expression of personality differs across interpersonal relationships. More in detail, participants in a study completed a five-factor measure of personality and constructed a social network detailing their 30 most important relationships. By leveraging contextual personality ratings they demonstrated the incremental validity when predicting specific informants’ perceptions. Indeed, variability in these contextualized personality ratings was predicted by the position of the other individuals within the social network. As a matter of fact, participants exhibited more extroverted and neurotic and less conscientious behavior, when interacting with central members of their social networks. Furthermore, dyadic social network–based assessments of personality provide incremental validity in understanding personality, revealing dynamic patterns of personality variability unobservable with standard assessment techniques.

In the last years the amount of data is continuously produced by different sensors (i.e. environmental or mobile devices) that need to be analyzed in real or near-real time by showing technical challenges and opportunities. A frequent itemset mining based on the Apache Spark Streaming framework has been developed by Fernandez-Basso et al. (2019) for extracting tendencies from continuous data flows using sliding windows. Ahmad et al. (2017) developed an approach based on an online sequence memory algorithm for anomaly detection on streaming data.

In the social media domain, a benchmarking of Big Data architecture using public cloud platforms has been discussed in Persico et al. (2018) for processing social streams. The learning-to-rank framework *Hashtagger+* (Shi et al. 2018) has been developed to analyze social streams in real-time by recommending hashtags to news articles. Furthermore, Wu et al. (2018) developed *StreamExplorer* based on sliding windows for visually exploring event in these streams. Social streams are also investigated in Zhang et al. (2017) for mining structural influence that has been defined as the combination effect of peer influences exerted by active friends on target users. Furthermore, the *InFlowMine* algorithm has been proposed by Subbian et al. (2016) for identifying information flows patterns.

## Conclusion

In this survey, we analyzed social networks research w.r.t. the big data paradigm dimensions. Our goal is to provide a quick guide through the plethora of published papers on the topic by following a direction tied to the different information carried out by each dimension of analysis. We covered the last two decades by mentioning papers that addressed big data features far before the big data paradigm took place. This survey can be used as starting point to navigate through the vast amount of papers published on SN when searching for some specific feature (e.g. volume). Obviously enough the concept related to some dimension received a higher attention by the research community (e.g. how to assess the Veracity of a news or how to extract gold nuggets from data), thus we tried to reflect this in the paper organization.

## Footnotes

1 https://www.scopus.com

2 Data gathered for the Global Digital Report

3 Those papers received many thousands of citations due to their relevance

4 Produced by Cambridge Intelligence software

5 At time of writing of this paper due to the COVID-19 virus emergency those statistics are more than ten times the reported typical values

6 https://en.wikipedia.org/wiki/Fakenews

7 http://www.snelgraphix.net/the-snelgraphix-designing-minds-blog/tag/google+I%E2%80%99m+feeling+stellar

8 https://www.businessinsider.com/banksy-arrest-hoax-2013-2
